# An exploration of students’ use of digital resources for self-study in anatomy: a survey study

**DOI:** 10.1186/s12909-023-04987-7

**Published:** 2024-01-09

**Authors:** Anna Pettersson, Klas Karlgren, Hans Hjelmqvist, Björn Meister, Charlotte Silén

**Affiliations:** 1 Department of Neurobiology, Care Sciences, and Society, Division of Physiotherapy, Karolinska Institutet, Solna, Sweden; 2 Department of Learning, Informatics, Management and Ethics, Karolinska Institutet, Stockholm, Sweden; 3 Department of Research, Education, Development and Innovation, Södersjukhuset, Stockholm, Sweden; 4 Faculty of Health and Social Sciences, Western Norway University of Applied Sciences, Bergen, Norway; 5 School of Medical Sciences, Örebro University, Örebro, Sweden; 6 Department of Neuroscience, Karolinska Institutet, Stockholm, Sweden

**Keywords:** Anatomy, Digital resources, Education, Learning, Self-study, Technology-enhanced learning

## Abstract

**Background:**

The development of technology has provided new ways for active engagement and for visualizing structures in anatomy education including digital resources that may be used outside of the classroom. To support students’ learning, there is a need to better understand students’ experiences of using digital resources. This study aimed to identify which resources students use, their preferences, the purpose of using them, and barriers to adopting tools for self-study of anatomy.

**Methods:**

A mixed -methods approach combining qualitative and quantitative data was used to collect and analyse data. Two consecutive cohorts of first-semester medical students (n = 278) were invited to complete an anonymized survey. The survey consisted of itemized questions, free-text space for comments, and one open-ended question. Descriptive statistics were used for demographics and itemized answers. Comments and free-text answers were analysed qualitatively using abductive inference.

**Results:**

One hundred and twenty-seven students completed the survey (response rate 45%). Most students (46%) reported that they spend more than 30 h/per week on self-study. They used a variety of digital resources for different purposes. Most students used digital resources to prepare for examinations, when they encountered difficulties and after going through a section. Students reported that they would use digital resources to a greater extent if they were offered an introduction, if resources were more accessible, and if they could interact with a tutor. The free-text responses revealed that digital resources helped students understand anatomy, allowed them to make active choices, provided tools for repetition and memorization, accelerated and simplified the learning process, and complemented other learning resources.

**Conclusions:**

Digital resources may support the understanding of anatomy by offering alternative modes of learning and providing a valuable complement to other learning resources. Educators should consider how digital resources are introduced and offer support and feedback.

**Supplementary Information:**

The online version contains supplementary material available at 10.1186/s12909-023-04987-7.

## Background

The development of new technology has fundamentally changed anatomy education, providing new ways for active engagement with content and for visualizing anatomical structures (1). In the literature, the term Technology-Enhanced Learning (TEL) is commonly used to describe the use of information and communication technology that enhances teaching and learning [1]. As the quality of TEL resources has evolved, there are greater opportunities for students to use digital resources outside of the classroom [2, 3]. This development combined with less time allocated to anatomy teaching, lack of cadavers, and larger student cohorts with a different mindset towards technology [4–7] as well as the recent pandemic has also pushed educators to use distant modes to deliver anatomy education [8–10]. Clunie et al. studied evaluation approaches used to assess the impact of TEL within anatomy education and showed that most studies reported learner satisfaction followed by effects on learning outcomes at the module or course level [11]. In addition to studies comparing different ways of delivering anatomy education [12–17] or comparing traditional methods with new ones [18, 19], there is a need for studies focusing on what students do that affects learning [20, 21]. Such studies can help educators better understand, not only what works but how and why. In two recent studies, we used an interactive tabletop to present 3D visualizations of real bodies, contributing to the knowledge of how students construct their understanding and interpret 3D images of topographical anatomy [22, 23]. One of these studies showed that what was significant for students’ understanding of images of anatomical structures was to let students discover images in their own way and pace and that students deliberately sought ways to create variations to fully discern the images, particularly regarding parts in relation to the whole of the body [22]. The other study reported on tutor and student perceptions of learning anatomy after a session of exploring anatomical images [23]. The result suggested that the use of authentic images was important for a sense of interest and meaningfulness, that students need time to explore on their own but also guidance and support, and that a mix of learning resources was perceived as more helpful than a single learning resource [23]. Another reason for studying how students learn is that a student-centred approach to learning has gradually gained traction within higher education and has changed the way learning is understood and organised [3, 24, 25]. Student -centred learning means shifting the focus from what teachers do to what students do [24]. Strategies to support students to take responsibility and direct their own learning have therefore become important [24, 26]. For learning to occur, students, themselves must actively study, experience, and construct their understanding, skills, and behaviour, and they must take responsibility for what and how they study [27]. They also need to understand their own learning processes and how to identify and apply different resources [26]. Previous experience and knowledge are important and will shape student learning. In addition, learning is not something that takes place in isolation, independent of the artifacts that students engage with. Recent research has increasingly emphasized that digital technologies are not only used to acquire information or for communication, technologies also provide a form of mediation. Paavola et al. suggested four different types of mediation in the context of educational technology [28]. First, epistemic mediation concerns creating and working with knowledge artifacts, including organizing and linking information. Second, technology can also support pragmatic mediation, which relates to organizing, planning, and coordinating the processes of knowledge creation. Third, social mediation concerns building communication through networks, social relations, and connections between different groups and communities to advance knowledge. Fourth, technology can provide reflective mediation, meaning it can make knowledge practices visible and contribute to their transformation [28].

Research investigating resources for learning anatomy on the Internet has mainly focused on Facebook, Twitter, and YouTube [6, 29–34]. Today, the range of anatomy resources available on the Internet is extensive, and it is not only limited to social media. However, research about how students perceive and use such resources is scarce, and there is a need to better understand how such resources may support students’ learning in their self-study.

The present study aimed to understand students’ experiences of using freely available digital resources in their self-study of anatomy. More specifically, the aim was to identify which resources they use, their preferences, the value they assign to preferred resources, the purpose of using them, and barriers to adopting such tools. A better understanding may help educators to identify strategies to support student self-study as part of self-directed learning. Here, self-study refers to the time students spend outside the classroom, during which students direct their own learning.

## Methods

### Research approach

In this study, we used a mixed-method approach by collecting and combining quantitative and qualitative data to reach a comprehensive understanding of students’ self-studies in anatomy [35]. The research approach assumes the existence of multiple realities where knowledge is constructed and shaped in a social world [36]. In line with this assumption, an interpretive approach was used to understand the phenomenon of learning anatomy during student self-study using freely available digital resources [36]. In this case, the researchers interpret the meaning of the data and are not considered objective. Credibility is maintained by including different perspectives and applying reflexivity and theoretical explanations in the research process [37].

### Context

The study was conducted at Karolinska Institutet, a medical university in Stockholm, Sweden. The medical program at Karolinska Institutet is a six-year programme (360 ECTS credits), leading to a license to practice. The teaching is based on a team-based learning (TBL) model, where students develop central skills, such as cooperation, communication, and leadership along with theoretical content [38]. The model consists of structured steps with preparatory work, small group learning, tests (readiness assurance test; RAT), feedback, and application sessions [38]. Within the framework of the study, dedicated time for self-study was included in the schedule. The study was performed after the completion of an anatomy module, which was part of a 12-week basic science course that focused on anatomy, histology, professional conduct and learning, clinical consultation, and physical examination. In addition, the course offered laboratory sessions with dissections, plastic models, and tutor-led demonstrations of real bodies using tabletop visualisation with 2-dimensional and 3-dimensional images. Students had free access to the Visible Body courseware (Visible Body, Newton, MA) through the university library. In addition, a link to 3D Slicer (an open-source software for the visualization of images) was provided via the learning management system Canvas. Images for visualization were available through Embodi3D®.

### Participants and data collection

Two consecutive cohorts of first-semester medical students (n 278) were invited to complete an anonymized survey related to their use of digital resources in the self-study of anatomy. The survey consisted of 10 itemized questions and one open-ended question, covering demographic data, type of digital resources, frequency of use, and purpose of use, as well as the students’ thoughts about the respective digital resources, how they used them in their study of anatomy, and perceived barriers to the use of digital resources (Appendix 1). The survey allowed multiple responses to the itemized questions, provided free-text space for comments, and included one open-ended question. The response options for the itemized questions were developed based on findings from our two previous studies with observational [22] and interview-based [23] data about students’ learning of anatomy. The open-ended question allowed for capturing other types of mediation as suggested by Paavalo et al. [26] The survey was distributed through a learning platform upon completion of a four-week anatomy module and after the students’ written exam. Reminder e-mails were sent on three occasions.

### Analysis

Descriptive statistics were used to summarize and present demographics and students’ answers to itemized questions (Appendix 1). Comments and free-text answers allowed a better understanding of itemized answers and were analyzed in two ways. Comments that could clarify or provide a good illustration of an itemized question were analyzed to that question. Comments that contributed to the understanding of students’ experience of using digital resources in their self-study of anatomy were analyzed together with free-text answers using qualitative content analysis inspired by abductive inference for interpretation of data [37, 39]. This is a dialectic and reflexive process where empirical findings are analyzed inductively alongside previous theories, results, and current understanding to generate plausible insights. The written texts were read several times and meaningful units related to students´ accounts about learning using digital resources were identified. The meaning units were condensed, interpreted abductively, and finally described as themes. Two of the authors (AP, CS) conducted the analysis and the procedure and themes were discussed with all authors reaching consensus.

### Findings

The findings are presented in two parts. The first part contains demographics and the main results of the itemized answers. Complete answers are presented in figures and in Table 1. In general, survey questions allowed multiple answers and free text comments. Quotes from comments are used to further illustrate the findings. The second part presents the results of the qualitative analysis of the open-ended question and free-text comments that shed light on the research questions.

### Demographics and itemized answers

One hundred and twenty-seven students (53% women; mean age, 23.4) (SD 5.6) completed the survey, which is equivalent to a response rate of 45%.

### The number of self-study hours

**Fig. 1:**
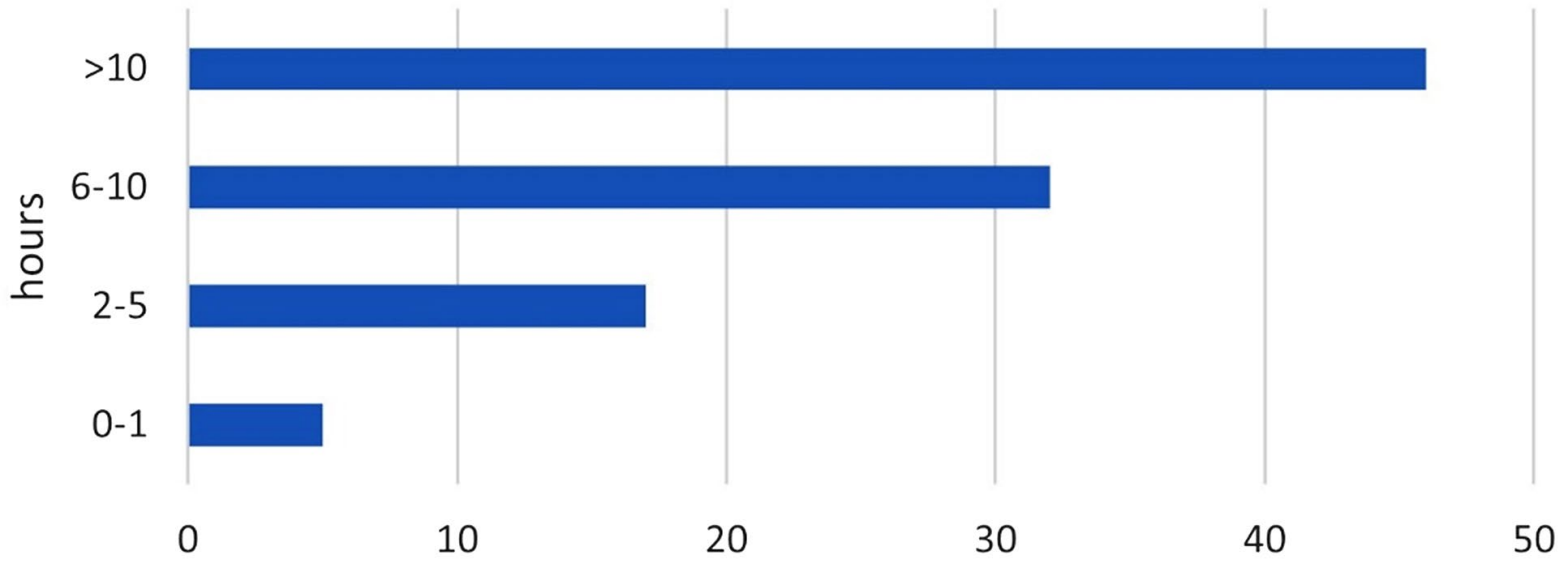
Q3. How much time have you used for self-study in the anatomy section on average per week? Proportion of students in %

The most common (46%) response among students was that they dedicated more than 30 h per week to self-study (Fig. 1).

### The digital resources used

**Fig. 2:**
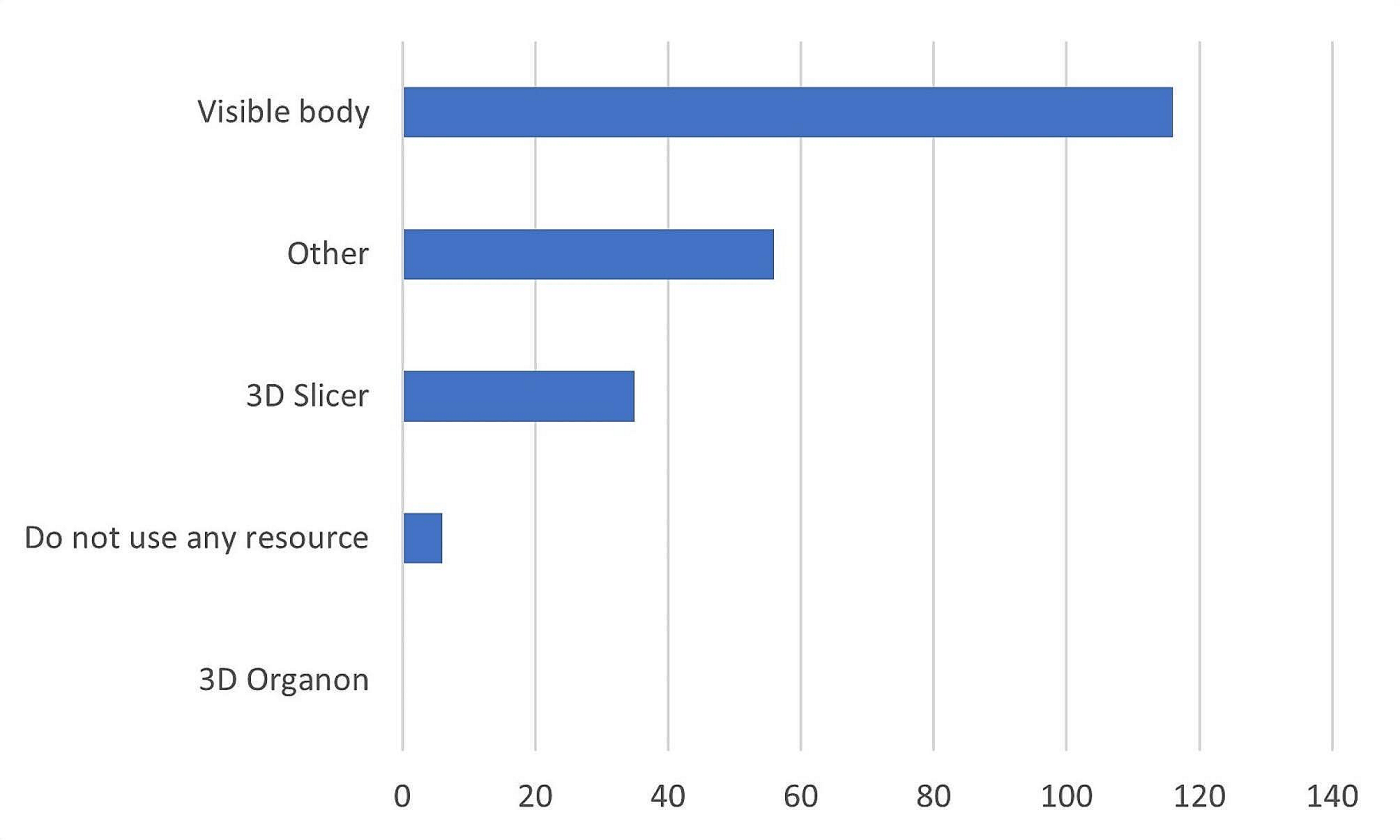
Q4. What digital resources for visualization do you use in your self-studies to learn anatomy? Multiple answers were possible

When asked what resources they use, students provided a range of answers (n = 212). The three most used resources were Visible Body, followed by “Other resources” and 3D Slicer (Fig. 2). Comments showed that several other (n = 8) resources were used, with Anki decks (a group of flashcards that show information for memorization) being the most frequently used resource in these responses (see Table 1 for an explanation of other resources). Of the resources that the students used, a majority of students ranked Visible Body as the most beneficial (51%), followed by 3D Slicer (40%) and “Other resources” (19%).

**Table 1:** Resources reported to be used by students, in alphabetical order.* Indicates a named source in the questionnaire. ^+^ Indicates a tool freely available by the university. The other resources presented were mentioned by students in the comment box

RESOURCES USED BY STUDENTS	DESCRIPTION
3D ORGANON*	A digital platform offering software solutions such as virtual reality anatomy based on models. Can be applied to different devices. © 2023 Medis Media
3D SLICER*	Free and open-source software for image analysis and visualization https://www.slicer.org/
ANATOMY AND DISSECTION CLUB	A student community at the University of British Columbia offering activities and networking within anatomy dissections. ® 2023 Anatomy and Dissection Club
ANATOMY ZONE	A UK-based website offering video tutorials on 3D anatomy models. © 2023 AnatomyZone
ANKI DECKS	An open-source software program that helps you create and synchronize flashcards for remembering across different devices. It is independent of content. There are online marketplaces for buying/selling premade flashcards within anatomy.
COMPLETE ANATOMY	A 3D anatomy software by Elsevier that provides 3D anatomy tools and materials (images, photos, models) to students, professionals, and/or institutions. ©2023 3D4Medical
DISSECTIONMASTERX	An application that provides digitalized, high-resolution images of human dissections to virtual reality. ©2023 Meta
DOCCHECK	A German digital community of medical professionals. As a member you can access videos, news, CME activities. ©Doccheck community GmbH
KEN HUB	An online platform offering anatomy learning tools and experiences that are validated by an international team of medical professionals. ©2023 Kenhub
NOTED ANATOMIST	A Youtube channel with videos and tutorials created by a Dr Morton. Dr Morton teaches anatomy to health care professionals but is not a medical doctor (affiliated to Utah university)
VISIBLE BODY* ^+^	An anatomy application by Wolters Kluwer that supports anatomy and biology learning and education resources in 3D for practitioners, educators and students. ©2023 Visible body

#### When the resources were used

**Fig. 3:**
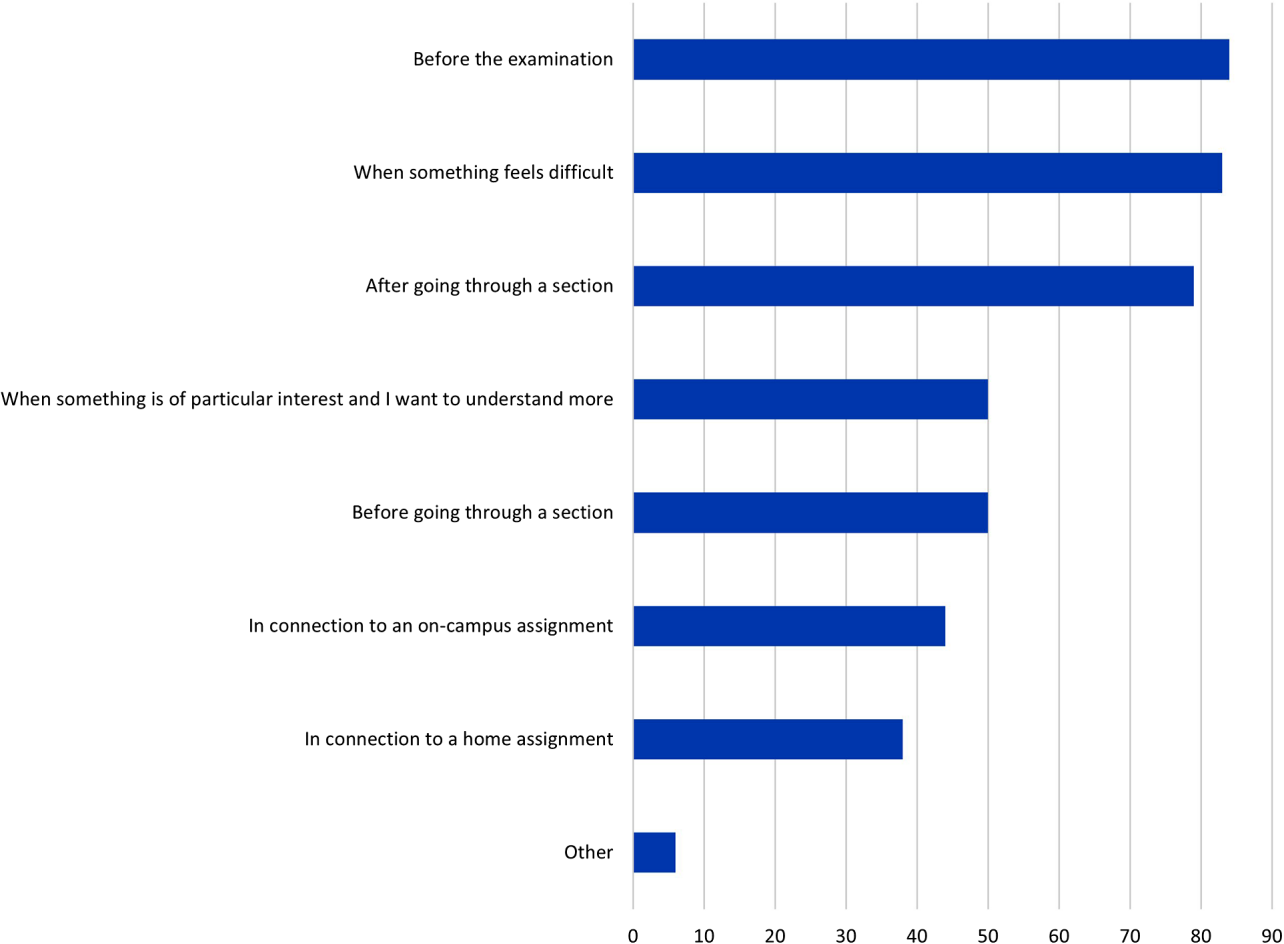
Q5. When do you use the digital visualization resources during your self-studies? Multiple answers were possible. Proportion of students in %

The resources were used at different time points during the course and students provided several answers to this question (n = 542). They were frequently used before the examination (84%), when something was perceived as difficult (83%), and after going through a particular Sect. (79%) (see Fig. 3 for a complete overview). The free-text comments (n = 19) provided additional insights into when students used digital resources for visualization.

“I used the visualization resources before some dissection to find structures more easily.”

“[I] often use Visible Body during lectures to visualize the material better”.

“Mostly as an addition to textbooks and quantity training using Anki [flash cards] to check that I can apply my knowledge ‘sharply’”.

### Reasons for not using digital resources

**Fig. 4:**
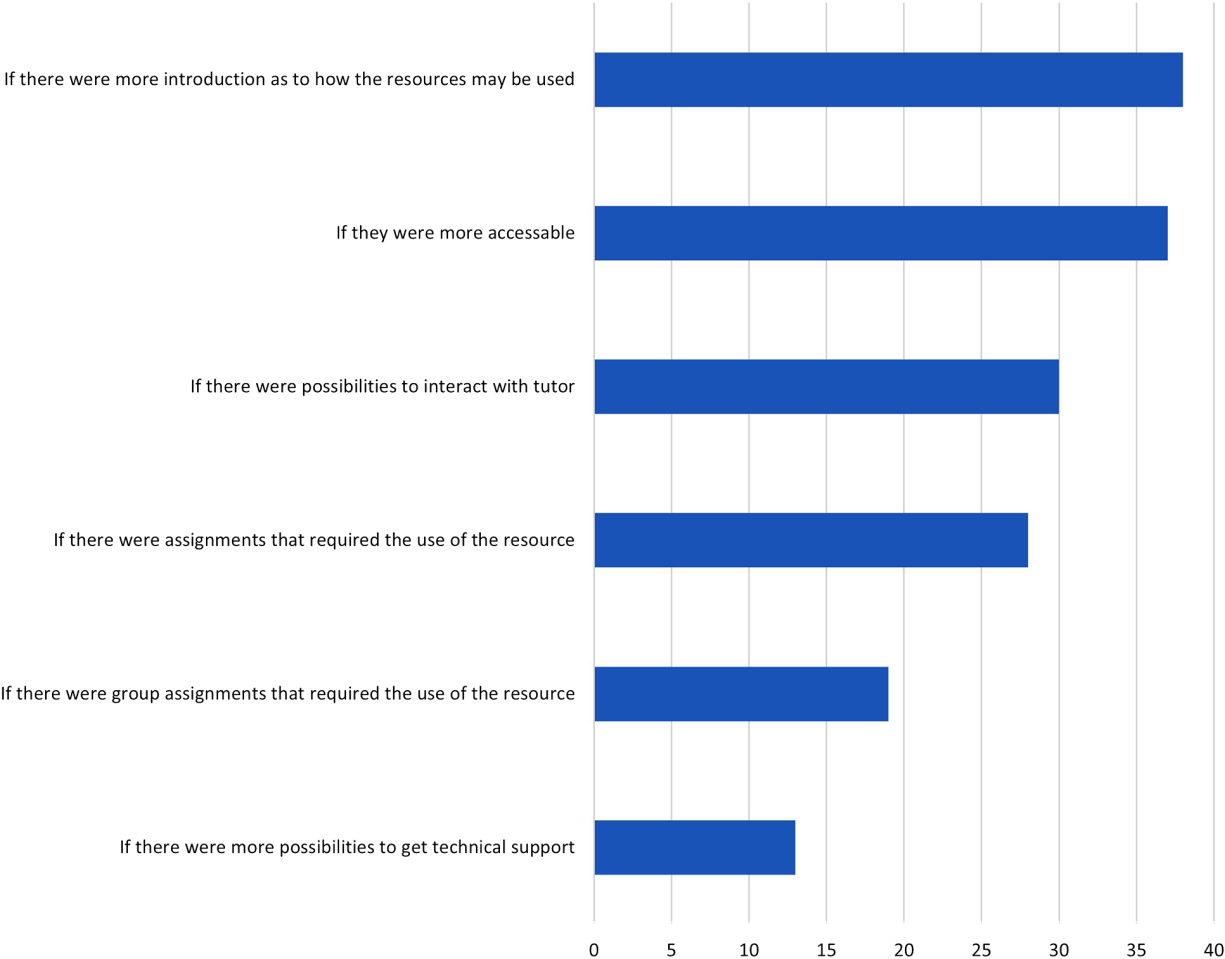
Q6. Reasons for not using digital resources

Some students reported that they did not use digital resources for visualization in their self-studies (24%). Among these students, the most common reason reported was that the anatomy books were sufficient (Fig. 4). The comments revealed that reasons for avoiding a resource were that the computer capacity was not sufficient, that the resource required the student to install an application on their mobile phones, or that they were not satisfied with a particular feature.

“They are heavy on the computer and cause the computer to lag. Therefore, it sometimes feels clumsy and not so effective”.

“Mainly used flashcards; Visible Body has written all structures in English instead of Latin – difficult!”

### Conditions that would make the students use the resource more

**Fig. 5:**
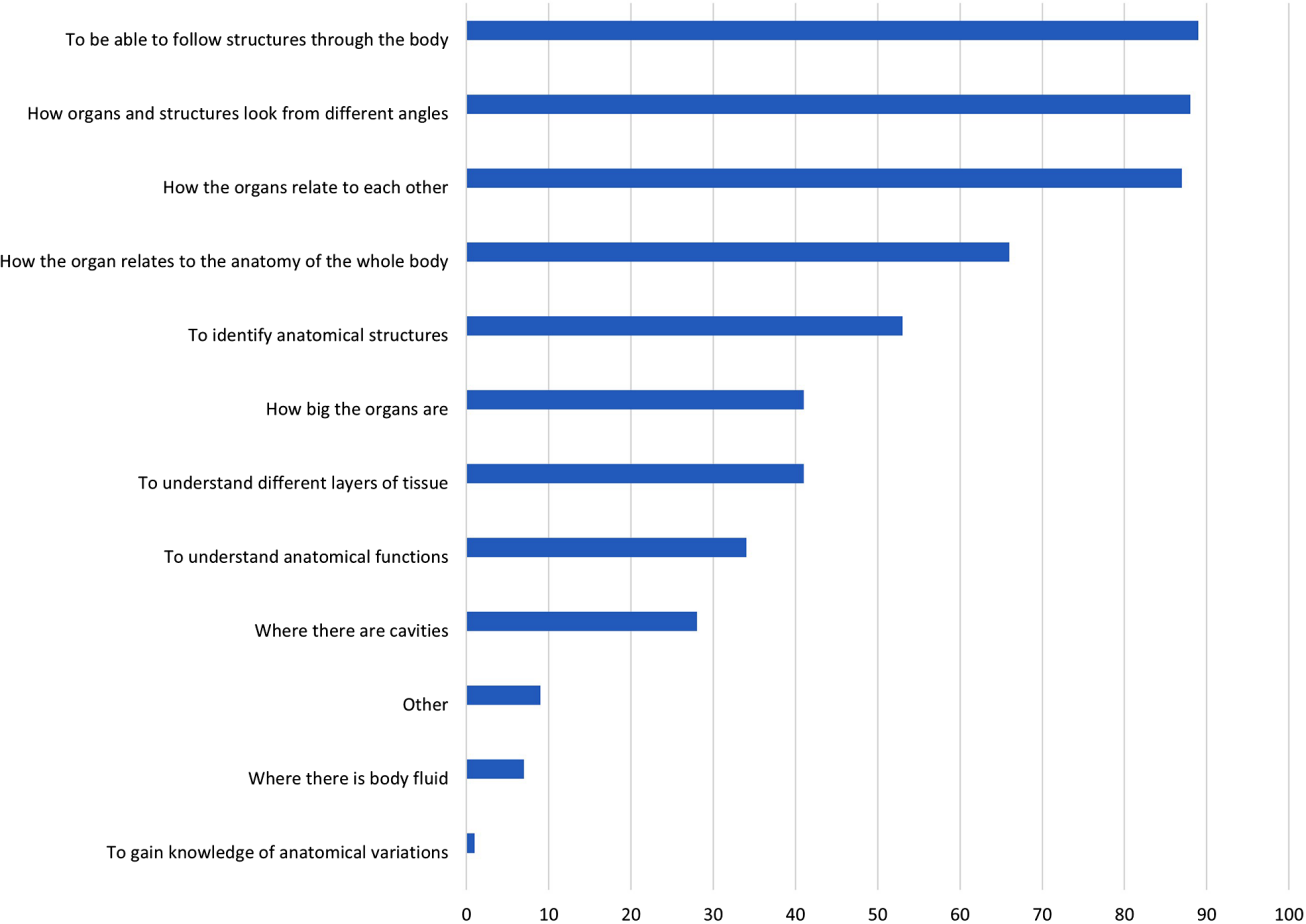
Q7. What would make you use digital visualization resources more in your own studies? Multiple answers were possible. Proportion of students in %

Students reported however that they would use digital resources more if an introduction was provided for the use of these resources (38%) and if resources were more accessible (37%). Furthermore, students reported that they would use digital resources more if they could interact with a tutor (30%) (Fig. 5).

### The added values of using digital resources compared to traditional resources

**Fig. 6:**
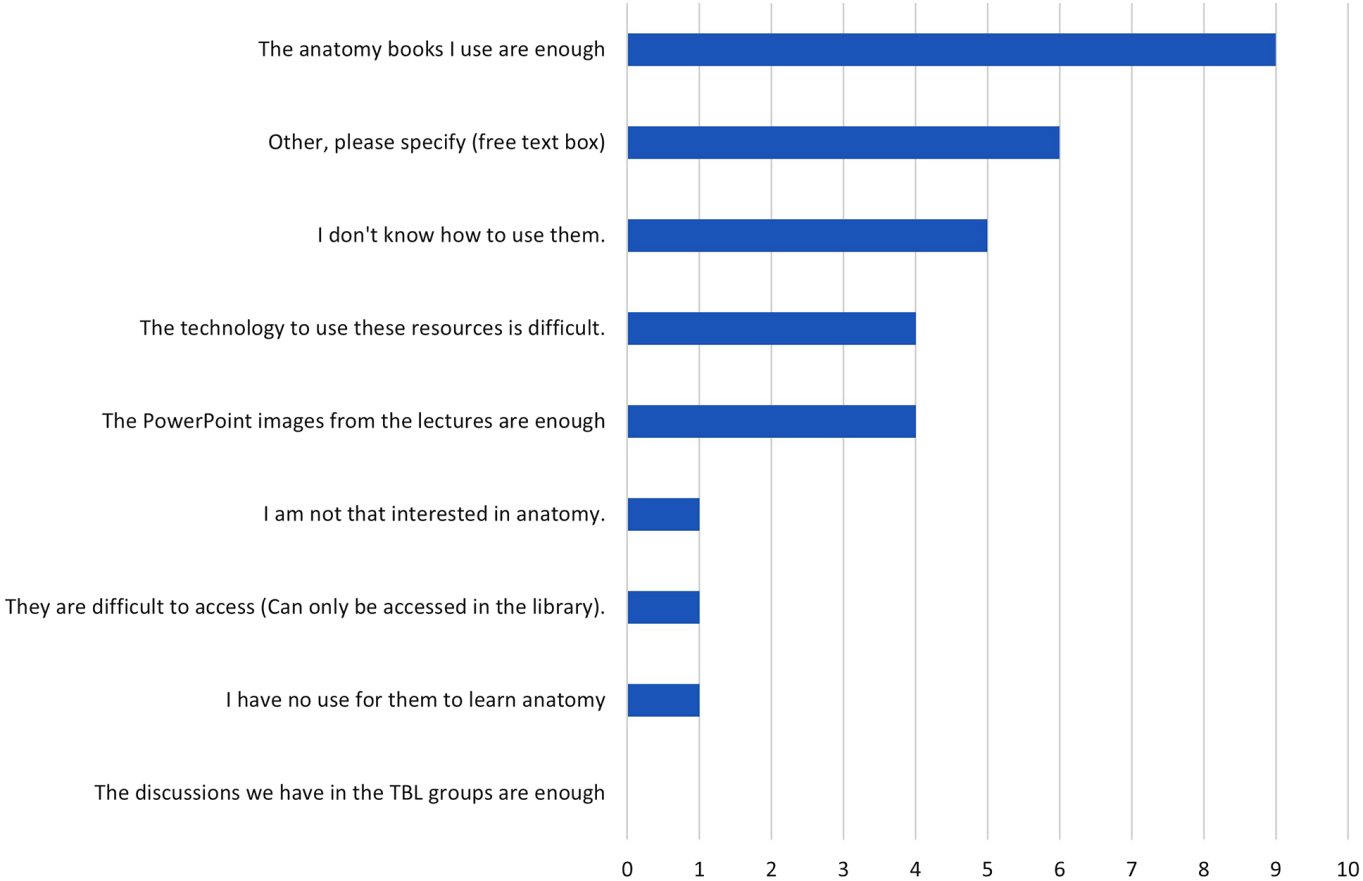
Q8. What added value do digital visualization resources have compared to, for example, an anatomy book/atlas, lecture or other teaching? Mulitple answers were possible. Proportion of students in %

The students who used digital resources for visualization in their self-study reported that these resources added value in a variety of ways compared to traditional learning resources (n = 691). For instance, digital resources allowed them to follow anatomical structures through the body (89%), to view organs from different angles (88%), and to see how organs are placed in relation to each other (87%) (see Fig. 6).

### How the digital resource was used

**Fig. 7:**
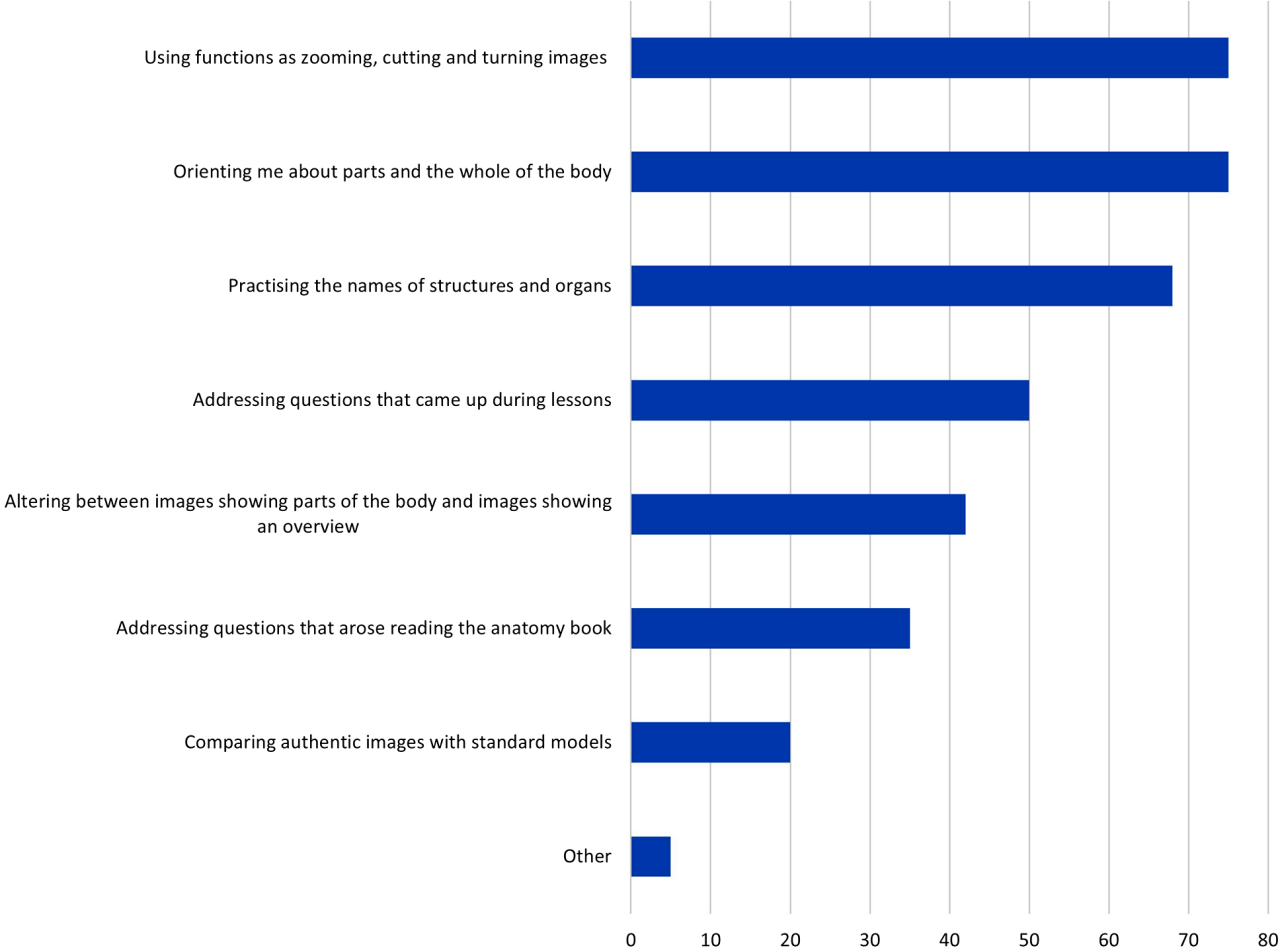
Q9. How do you use the resources? Multiple answers were possible. The proportion of students in %

Students provided 469 answers about how they used digital resources. These resources were used to interact with images, (75%), orient the student to the parts and the whole of the body (75%), and learn the names of structures and organs (68%) (see Fig. 7 for a complete overview).

### The extent to which the digital visualization resource was helpful in passing the examination

**Fig. 8:**
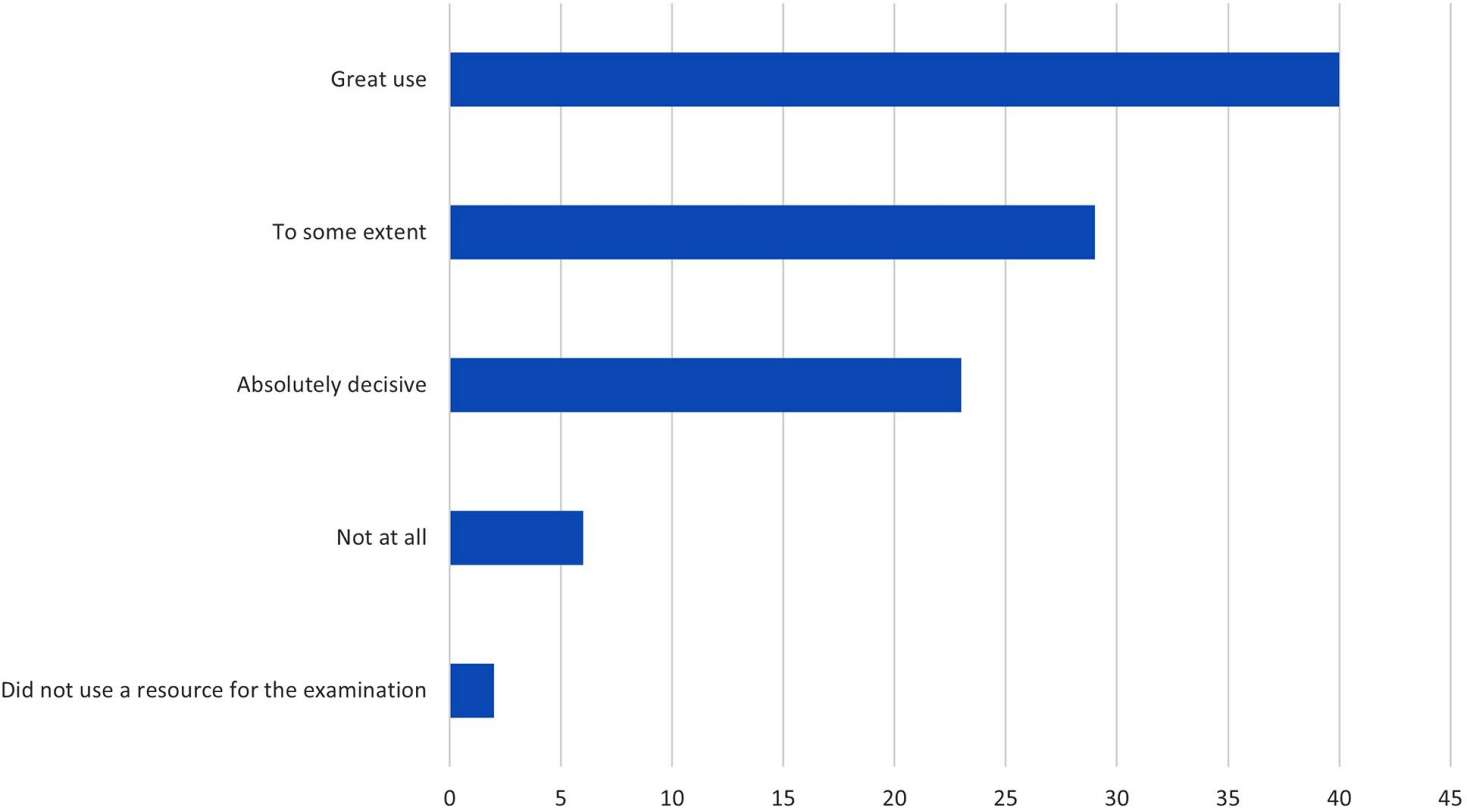
Q10.Did you benefit from using digital visualization resources in your own studies to pass the anatomy exam? Proportion of students in %

40% of the students reported that digital visualization was extremely useful in preparation for the examination, 29% reported that it offered some benefit, and 23% of the students thought it was the most valuable tool. A small proportion of students did not find digital visualization to be beneficial at all (6%) or had not used it to prepare for examinations (2%) (Fig. 8).

“I have used it sometimes when it has simplified the work, but I could probably have got by with only Petrén as well [a classical Swedish anatomy book] if I wanted to. People have done that before”.

“Digital visualization resources for studying the human body are, in my opinion, absolutely phenomenal and groundbreaking. The students who were not part of the ‘old days’ before these probably cannot understand how well off they are regarding this”.

### Qualitative analysis of free text answers and comments

In addition to pre-formulated answer options, the students were invited to express their experiences of using the digital resources they used the most. In this second part of the findings, we present data from the open-ended question and free-text answers from comment boxes with relevance to the research question. Data were collated and qualitatively analyzed using an interpretative abductive approach as previously described [37].

**Fig. 9:**
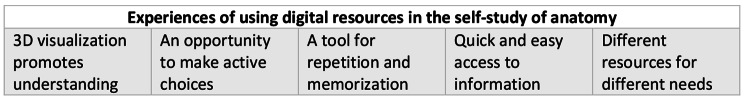
Experiences of using digital resources

Students’ comments on their use of digital resources in their self-studies supported as well as added information to the itemized answers. Digital resources could contribute to their understanding of anatomy, but they also expressed benefits related to opportunities to plan and use the resources in relation to their own needs. Five main categories displaying different aspects of students’ experiences were identified from the data (Fig. 9). Categories are explained, and illustrative quotes are presented below.

### 3D visualization promotes understanding

Digital resources providing three-dimensional images seemed to contribute to a deeper understanding of anatomy. The students brought forward that resources with 3D images could be used to identify what a particular anatomical structure looks like and how it is related to other structures and organs. Engaging with visualization presented as 3D offered them a greater sense of the body anatomy as a whole in relation to the parts and offered them an overview.

“You get an overview of how things are”.

“Visible Body is good for understanding how structures transcend into/relate to other structures”.

### An opportunity to make active choices

An important dimension of using digital resources was expressed as having the opportunity to decide for yourself how the actual resource should be used. The students highly valued the opportunity to take an active role in their learning by adding and removing layers of tissue and being able to control which structures they wanted or needed to study more in-depth.

“That you can add/remove exactly the structures you want, so you can only follow what you need to check”.

“[it provides the] possibility of deepening by being able to remove structures and look at certain types of organ systems in isolation”.

### A tool for repetition and memorization

It became clear that students also appreciated digital resources that offered them help to hammer in anatomy knowledge. Students reported consistently that an advantage of flashcards was that they helped them memorize the terminology and learn the location of anatomical structures.

“[I]use flashcards from Anki, self-made or by a fellow student to regularly grind in the names and locations of anatomical structures. [It’s a] good way to get spaced repetition and thus get something from short-> long-term memory”.

“Anki is of the utmost importance for memorizing knowledge for me and my fellow students”.

### Quick and easy access to information

The students described efficient access to informative learning resources as a valuable factor in their learning. Quick and easy access to information and the functionality of the resources became important to fulfill the requirement of efficiency and learning. They especially valued finding multiple functions in one place, clear and unambiguous information, and the combination of images and explanations.

“In Visible Body, everything is 3D, and it tells you what each structure is called. If it is a muscle, you also get to know innervation and function. On many structures, you can also press a button to read more about them”.

“In Flexikon from Doccheck (in German), almost all anatomy is described, sometimes with associated videos or 3D models. For vessels, you can see the original vessel, but also which branches come for that vessel. Finally, Flexikon contains very good explanations and descriptions of almost all anatomical structures that we need to know”.

“[It’s good] that you can see good information about the structure you have marked on in the Visible Body. With [3D] Slicer, I wish it had a little more info than just pictures so you can know for sure what you’re finding”.

### Different resources for different needs

The students’ comments and free-text answers revealed that they used strategies in their studies of anatomy. They put forward that a variety of resources, both traditional and digital, were needed to learn. Specific purposes guided their choice of learning resources, and they used them strategically (i.e. in a certain order) in their studies.

This was expressed in terms such as “First I do this and then I do (…)”, or “When I want to…, I use (…).”. In this way, the resources complemented each other and met different needs in the students’ self-study.

“First, I used flashcards to memorize structure names, shapes, and locations. Then I used video lectures on YouTube to understand the relationships between the structures. Both dissection reviews from UBC and video lectures from Noted Anatomist”.

“The book goes a long way, I think, and is better in certain respects. But Visible Body has been great for quickly finding a structure you are looking for, being able to follow it and see how it is placed in relation to other things in a way that had taken much more time to find out in the book”.

“[I] usually start from the lecture first to have a certain overview of the area before starting to study. I only use Visible Body if I want to look up a specific structure, [if] I’m not sure exactly where it is, and how it is in relation to other structures. The coursebook is mainly used to process and learn, while the Anki is used to rehearse and memorize the names of the structures”.

## Discussion

This study set out to understand how students use digital resources in their self-study of anatomy and to determine preferences, purpose, and barriers to the use of these tools. The results provide new insights into the kind of resources students use, as well as how they use them for learning. This has implications for how teachers should introduce digital resources in anatomy courses and how they can best support self-directed learning. Previous research on digital resources has studied the use of social media, such as Facebook and YouTube [6, 29–34]. Some authors have suggested that although social media can be a valuable part of anatomy education, faculty should engage with social media and consider integrating it into formal teaching to ensure its correctness and adequate use [6, 31]. The findings of this study show that students accessed and used a wide range of TEL resources in their self-study and that these resources supported their understanding of anatomical structures, such as different layers of tissue, how structures relate to each other, or the size of organs. This active strategy for learning is in line with previous findings from observational [22] and interview-based studies [23], as it emphasizes the active involvement of the student. At the same time, the students’ answers suggest that they would use the digital resources more if they had received an introduction as to how to use them, if the digital resources had been more accessible, and if students had the opportunity to interact with a tutor. Research about self-directed learning has shown that students need facilitation and support to gain confidence in their self-study practices [26]. Jaffar (2012) argued for the introduction of social media in anatomy education based on the reasoning that today’s students “grew up in an environment enriched by information technology” and that the use of the Internet has become an integral part of everyday life [30]. The results of the present study suggest that an introduction and support in the use of TEL can still be appreciated and useful for many students. The findings from this study and previous research [22, 23] suggest that learning should be arranged to allow students to direct their own learning and allow time for an active discovery process while providing teacher-student/tutor-student interaction to offer feedback and support. Such feedback and support could be about confirming the student’s interpretation, hypothesis, or strategy, answering questions, helping students make connections to the clinical setting, or providing technical support in terms of “how-to” resources. This can promote student motivation and help them move forward in their learning process [23, 26]. This could very well have been integrated into the TBL model that was used for the students in the studied context.

In addition to the digital resources that were introduced in the course (Visible Body, 3D Slicer, and 3D Organon), the students in this study reported that they used eight other resources. “Other resources” were the second most used resources as reported by the students. The reported resources were websites and applications specifically designed for anatomy education, which offered solutions for virtual reality, tools for visualization, quizzes and tests, and networking activities. In terms of the different resources reported, most originated from professional communities or companies within TEL, education, and research.

In a review from 2019 that looked at the use of social media in anatomy education, the author concludes that the studies carried out so far originate from a limited number of countries (four out of nine studies were carried out in the Middle East) [32]. As research from a variety of cultural and educational contexts may provide a more meaningful understanding of teaching and learning across educational settings, the present study adds to the existing body of research by presenting a Scandinavian perspective.

As presented in the introduction, technology fosters different kinds of learning processes [28]. Although the survey referred to students’ self-study, it was interesting that few answers and no comments related to social mediation, as the studies took place in a TBL context that required collaboration. Another characteristic of TBL is that students prepare for teaching and learning activities [38]. This was also not reflected strongly in the findings. A curriculum with organized group activities creates a range of opportunities for discussion and for students to learn from each other, not only what students learn using TEL, but how they use these resources to support the development of self-directed learning [26]. Most of the students’ comments referred to epistemic mediation, which reflects the student’s individual learning [28]. When students were asked when they used a particular resource, the most common answer was to prepare for the examination when something was perceived as difficult, or after going through a section. TEL resources also did not seem to foster pragmatic mediation, reflecting students’ attempts to plan and organize knowledge creation [28]. Some of the students who used quizzes to rehearse and some who used Anki decks may have actively chosen and created questions purposefully according to their own specific learning needs. However, most students seemed to use ready-made Anki decks, which were adjusted to include questions commonly used in the written exams.

The number of answers provided by the students and their comments indicate that students use TEL resources for different purposes and that they fulfill different needs. Some resources were used for memorization and others were used to grasp the whole in relation to the parts, the 3-dimensional aspects, and the relationship between anatomical structures. Answers and comments indicate that this was an active and deliberate process and that students often used the resources in a particular order. Panday and Zimitat found that students who perceived anatomy learning to be difficult and used a combination of memorization, understanding, and visualization had better final grades [12]. Darras et al. point out that using virtual dissection of patient CT scans, in combination with traditional dissection, adds further dimensions in that CT scans of patients can provide a kind of learning that is not possible with traditional dissection alone (e.g., bowel gas patterns) [14]. Virtual dissection, on the other hand, does not provide the same tactile experience. Somewhat contradictory to this is that when students were asked about the value of TEL resources in comparison to traditional ways of learning, the opportunity to view actual body fluids or cavities was not frequently cited as an advantage. This may suggest that students need support to explore anatomical structures in a meaningful way. A systematic review by Coyne et al. [40] looked at blended learning within anatomy education and showed that students appreciated the flexibility that blended learning provides. This suggests that it may be appropriate to stop comparing TEL resources to traditional dissection and to focus on exploring how anatomy education can be enhanced by using combinations of resources and strategies and how educators can support students’ self-directed learning.

### Limitations

An adequate sample size is necessary to reach valid conclusions; a response rate of 50% can be considered acceptable in survey research [41]. This study attained a response rate of 45% which is a limitation. However, the purpose of the study was not to make generalizations but to collect rich data to better understand how students use TEL resources in their self-study. Data came from two separate cohorts, which meant avoiding a snapshot of the phenomenon. Another concern could relate to the items and response alternatives covered. The chosen items and response alternatives were developed based on findings from two previous studies, which likely improve validity. One open-ended question and free text comments allowed respondents to provide perspectives that were not captured by the pre-decided response alternatives provided. The anonymized survey sent out by mail presents a certain risk of bias towards certain respondents, favoring respondents with an interest in participation. The survey depended on retrospective reports which can cause a risk of memory bias when responding. On the other hand, if time passes, it might enable for more nuanced reflection and reframing of the learning situation. The responses and comments also reflected respondents who did not use digital resources in their self-study.

## Conclusions

According to students, a variety of digital resources are used in their self-study in anatomy, specific purposes guide their choice of learning resources, and digital resources provide opportunities for students to take an active role in their learning. The findings indicate that educators should consider how digital resources are introduced and offer support and feedback during the learning process to help students become self-directed in their learning. Further research is needed to explore how technology-enhanced learning resources are best combined and how feedback is used to enhance learning.

### Electronic supplementary material

Below is the link to the electronic supplementary material.

Supplementary Material 1

## Data Availability

The datasets analyzed during the current study are available from the corresponding author on request.
