# Supplementary material for: An exploration of students’ use of digital resources for self-study in anatomy: a survey study
Source: BMC Med Educ. 2024 Jan 9;24:45. doi: 10.1186/s12909-023-04987-7 (PMC10777562; doi:10.1186/s12909-023-04987-7)
Supplement: Supplementary file 1 — Supplementary Material 1 [file 12909_2023_4987_MOESM1_ESM.docx]

Appendix 1

**QUESTIONNIARE**

Q. 1 Please enter your age

Q. 2 Please enter your gender

Q.3 How much time have you used for self-study in anatomy on average per week? Please, tick the option that suits you best!

a) 0-1

b) 2-5

c) 6-10

d) > 10

Q4. What digital visualization resources do you use in your own studies to learn anatomy? Please, tick ​​the options that suit you best!

1. Visible body
2. b) 3D slicer
3. c) 3D Organon
4. d) Other resource (free text box)

Q5. When do you use these resources? Tick ​​all the statements that apply to you!

1. Before we go through a section.
2. After going through a section.
3. When we have been given an assignment to work on at home.
4. When we have been given an assignment to work on campus.
5. When I think something that is difficult to understand.
6. When I think something that is particularly interesting and want to understand more.
7. Before the station examination in anatomy.
8. Other, please specify (free text box)

Q6. If you are not using digital resources, we are interested in knowing why. Please, tick ​​all the statements that apply to you!

1. I have no use for them to learn anatomy.
2. The PowerPoint slides from the lectures are enough,
3. The anatomy books I use are enough.
4. The discussions we have in the TBL groups are enough.
5. The technology to use these resources is difficult.
6. They are difficult to access (Can only be accessed in the library).
7. I don't know how to use them.
8. I am not that interested in anatomy.
9. Other, please specify (free text box)

Q7. What would make you use digital visualization resources more in your own studies? Please, tick ​​all the statements that apply to you!

1. That they are more easily accessible.
2. More introduction to how the resources can be used.
3. Opportunities to get help with technical questions.
4. Opportunities to interact with the tutor if you have questions about the images.
5. Tasks that require use of resources.
6. If there were tasks to work on together in a group.

Q8. What added value do digital visualization resources have compared to, for example, an anatomy book/atlas, lecture or other teaching. Please, tick ​​all the statements that apply to you!

1. To identify anatomical structures.
2. To be able to follow structures through the body.
3. How organs and structures look from different angles.
4. How organs relate to each other.
5. How big the organs are.
6. How organs relate to the anatomy of the entire body.
7. Where there are empty spaces.
8. Where there is body fluid.
9. To understand different layers of tissue.
10. To understand anatomical functions.
11. To gain knowledge about anatomical variations.
12. Other, please specify (free text box).

Q9. How do you use the digital visualization resources? Please, tick ​​all the statements that apply to you!

1. Based on questions that came up during lectures.
2. Based on questions that come up when I read the anatomy book.
3. I orient myself about the whole and parts of the body.
4. Use functions such as zooming in and out, making cuts, turning organs and the body.
5. Switch between looking at pictures of parts of the body and more overall images.
6. Practice the names of organs and structures.
7. Compare resources with authentic images with drawn/standardized images.
8. Other, please specify (free text box).

Q10. Did you benefit from using digital visualization resources in your own studies to pass the anatomy exam?

1. Not at all.
2. To a certain degree.
3. Great benefit.
4. Decisively.
5. I haven’t used digital resources in my self-studies.
